# Central Nervous System Aspergillosis: Advances in Diagnosis, Therapeutics, and Multidisciplinary Management (2026 Update)

**DOI:** 10.1111/myc.70214

**Published:** 2026-09-07

**Authors:** Moustafa A. Mansour, Mohamed Wahid, Mohamed A. S. El Molla, Mohamed H. Helmy, Taha Mohammed Adel, Hamdi Nabawi Mostafa

**Affiliations:** ^1^ Department of Neurosurgery Nasser Institute for Research and Treatment Cairo Egypt; ^2^ Department of Neurosurgery Mayo Clinic Foundation Rochester Minnesota USA; ^3^ Department of Neurosurgery Misr University for Science and Technology Giza Egypt

**Keywords:** antifungal therapy, blood–brain barrier, CNS aspergillosis, diagnostic advances, immunomodulation, novel antifungals

## Abstract

**Background:**

Central nervous system (CNS) aspergillosis is a life‐threatening infection with mortality rates exceeding 50%, especially in immunocompromised patients. Significant challenges persist due to limited antifungal drug penetration into the CNS, emerging resistance, and diagnostic delays, despite advancements in therapy and diagnostics.

**Objective:**

This comprehensive review aims to synthesize pivotal advances in the management of CNS aspergillosis from 2020 to 2026 and to provide a multidisciplinary framework for addressing these ongoing challenges.

**Methods:**

We conducted a comprehensive evaluation of the latest clinical data, pharmacokinetic studies, and expert recommendations from the specified period. The review critically appraises evidence on pharmacological therapies, diagnostic technologies, and adjunctive treatment strategies.

**Findings:**

Key findings include: *Pharmacotherapy*: Voriconazole remains the cornerstone of therapy due to its superior CNS penetration (CSF:Plasma ratio ~50%). The roles of alternatives like isavuconazole, salvage combination regimens, and novel agents (e.g., olorofim, fosmanogepix) are evolving. *Diagnostics*: Cutting‐edge tools such as AI‐assisted imaging, metagenomic next‐generation sequencing (mNGS), and MR spectroscopy for trehalose detection show significant potential for enabling earlier and more accurate diagnosis. *Adjunctive Strategies*: Neurosurgical intervention, immunomodulation, and therapeutic drug monitoring (TDM) are critical for optimizing outcomes. Emerging strategies like nanoparticle‐based drug delivery and host‐directed therapies (e.g., PD‐1/PD‐L1 blockade) offer promising avenues to overcome the blood–brain barrier.

**Conclusion:**

This review integrates the latest evidence to provide a timely and actionable resource for clinicians. It bridges gaps in existing guidelines by offering a multidisciplinary approach that addresses the complex management of CNS aspergillosis, with particular relevance for high‐risk populations such as COVID‐19 and immunocompromised patients.

**Abbreviations**
CAPA: COVID‐19‐associated pulmonary aspergillosis
CEST: Chemical exchange saturation transfer
CNS: Central nervous system
CSF: Cerebrospinal fluid
ELISA: Enzyme‐linked immunosorbent assay
HSCT: Hematopoietic stem cell transplants
LA: Latex agglutination
mNGS: metagenomic Next‐generation sequencing
MR: Magnetic resonance
MRS: MR spectroscopy
TDM: Therapeutic drug monitoring

## 1 Introduction

Central nervous system (CNS) aspergillosis is a devastating fungal infection with extremely high mortality rates, particularly affecting immunocompromised patients. Recent epidemiological data highlight persistent challenges despite advances in therapy [1]. Specific risk factors include the use of BTK inhibitors, which have been associated with an increased incidence of CNS aspergillosis [2]. Despite advances in antifungal therapy, the overall case‐fatality rate remains around 58%, with rates as high as 86.7% in bone marrow transplant recipients and 88.1% in cases of CNS or disseminated aspergillosis [3]. Traditional treatments like amphotericin B have shown limited efficacy due to poor CNS penetration [1]. However, voriconazole, a newer antifungal agent with better CNS penetration, has demonstrated improved outcomes, with complete or partial responses in 35% of patients [4]. Neurosurgical interventions, when feasible, have been associated with improved survival rates compared to medical treatment alone [5]. While primarily affecting immunosuppressed individuals, CNS aspergillosis can also occur in immunocompetent patients, highlighting the need for improved diagnostic and treatment strategies [5]. While current international guidelines address invasive aspergillosis in general, none provide targeted recommendations for CNS involvement. This review aims to bridge that gap by summarizing pivotal advances between 2020 and 2026 in diagnostics, therapeutics, and multidisciplinary management.

## 2 Epidemiology and Risk Factors

### 2.1 Incidence and Trends

The incidence of invasive aspergillosis following hematopoietic stem cell transplants (HSCT), solid organ transplants, and patients with prolonged neutropenia or hematologic malignancies ranges from 0.5% to 3.2%, depending on the type of transplant [6]. CNS involvement occurs in approximately 3% of allogeneic HSCT recipients, with a median onset of 124 days post‐transplant. Improved survival in these groups may paradoxically increase the time window for opportunistic infections like CNS aspergillosis.

### 2.2 Emerging Risk Groups

Recent years have seen the emergence of new at‐risk cohorts. One notable group includes patients with COVID‐19‐associated pulmonary aspergillosis (CAPA). Several case reports and series have documented CNS dissemination in critically ill, ventilated patients [7]. Another important consideration is the use of immune checkpoint inhibitors, which can paradoxically disrupt immune homeostasis and predispose to opportunistic fungal infections, including CNS aspergillosis [8].

### 2.3 Pathogen Diversity

Recent studies highlight the increasing recognition of non‐fumigatus *Aspergillus* species in invasive aspergillosis (IA), emphasizing the importance of accurate identification and antifungal susceptibility testing [9]. Species such as *A. flavus* , *A. terreus* , *A. niger* , and *A. nidulans* often exhibit intrinsic resistance or elevated minimum inhibitory concentrations to standard antifungal agents, complicating treatment [10, 11]. *A. flavus* is the second most common species in IA, while *A. nidulans* is frequently associated with primary immunodeficiencies and disseminated infections [11]. Cryptic species within the *A. fumigatus* complex, such as *A. lentulus* and A. udagawae, are often misidentified and may display azole resistance [12]. Advanced diagnostic techniques like specific gene sequencing, multiplex PCR assays, and MALDI‐TOF MS are crucial for accurate species‐level identification [12]. Improved diagnostics and susceptibility testing are essential for optimal management of non‐fumigatus *Aspergillus* infections [9, 11].

## 3 Diagnostic Challenges and Advances

### 3.1 Imaging Techniques

CNS aspergillosis typically presents with solitary or multiple ring‐enhancing lesions on MRI, often with surrounding vasogenic edema, restricted diffusion, or hemorrhagic components [13]. These findings are, however, non‐specific and overlap significantly with other CNS infections, neoplasms, and demyelinating diseases.

Importantly, cerebral aspergillosis manifests differently in neutropenic versus non‐neutropenic patients. Neutropenic patients typically show minimal inflammation with poorly defined lesions, hemorrhagic infarcts, and sparse contrast enhancement, whereas non‐neutropenic patients more often develop well‐formed abscesses with rim enhancement and surrounding edema, reflecting differences in immune‐mediated tissue damage and fungal angioinvasion [1, 13].

Advanced imaging modalities such as perfusion‐weighted imaging and MR spectroscopy (MRS) have shown promise in distinguishing fungal abscesses from bacterial or neoplastic lesions, though their availability is often limited to tertiary care centers [14]. MRS has shown promise in differentiating fungal abscesses from other brain lesions. Multiple studies have identified a characteristic peak between 3.6 and 3.8 ppm in fungal infections, attributed to the presence of trehalose, a disaccharide found in fungi [15, 16]. This trehalose peak has been observed in various fungal infections, including Blastomycosis and Cryptococcus [15, 17]. The presence of trehalose can help distinguish fungal abscesses from pyogenic abscesses, malignant neoplasms, and gliomas [15, 16]. While some studies have found lipid signals indicative of necrosis in fungal abscesses [18], the trehalose peak remains a more specific marker for fungal infections. Chemical Exchange Saturation Transfer (CEST) MRI has also shown potential in detecting trehalose, offering high spatial resolution for identifying and differentiating cryptococcomas from gliomas [17].

### 3.2 AI‐Assisted Diagnostics

Artificial intelligence (AI) shows promise in diagnosing CNS aspergillosis and other fungal infections. AI‐based systems can distinguish between *Aspergillus* and Mucorales in histopathological images, potentially improving diagnostic accuracy [19]. Machine learning algorithms have been applied to predict mortality and treatment outcomes in CNS infections, offering potential for rapid diagnosis and early treatment [20]. AI could also enhance the analysis of CT imaging in chronic pulmonary aspergillosis, although challenges remain in developing dedicated imaging registries and harmonizing annotations [21]. Further research is needed to fully realize AI's potential in diagnosing and managing CNS aspergillosis.

### 3.3 CSF and Molecular Diagnostics

Cerebrospinal fluid diagnostics play a crucial role in detecting CNS aspergillosis. CSF galactomannan testing demonstrates high specificity (94.4%) but lower sensitivity (69.0%) for CNS aspergillosis diagnosis [22]. *Aspergillus* PCR in CSF shows promising results, with studies reporting sensitivities of 75% [23] and 100% [24], and specificities of 98.3% and 93%, respectively. These PCR assays may reduce the need for invasive cerebral biopsies in suspected cases. Another study found CSF‐PCR positive in all five patients with CNS aspergillosis, while conventional cultures were negative [25]. Enzyme‐linked immunosorbent assay (EIA) and latex agglutination tests (LA) on CSF samples also showed potential for diagnosis [25]. Additionally, emerging adjuncts such as CSF β‐D‐glucan and cytokine profiling may enhance diagnostic confidence but are not yet standard of care [26]. Metagenomic next‐generation sequencing (mNGS) of CSF has emerged as a valuable tool for diagnosing CNS infections, including cerebral aspergillosis. Studies have shown that mNGS demonstrates high sensitivity and specificity in detecting *Aspergillus* species in CSF, with one study reporting 85.71% sensitivity and 84.62% specificity [27]. mNGS can accurately identify multiple *Aspergillus* species and co‐pathogens, potentially guiding targeted antimicrobial therapy [28]. In a large prospective study, mNGS showed varying performance across different CNS infections, with 80% sensitivity for cerebral aspergillosis [29]. The technique has proven particularly useful in diagnosing atypical pathogens and understanding the clinical presentation of CNS *Aspergillus* infections in both immunocompromised and immunocompetent patients [30]. These findings suggest that mNGS is a promising diagnostic tool for CNS aspergillosis, potentially reducing the need for invasive procedures like cerebral biopsies. However, the associated high cost, limited availability, and risk of contamination are major limiting factors for mNGS applications.

A critical clinical consideration is the impact of prior antifungal therapy on test sensitivity. Empirical antifungal treatment, particularly with voriconazole or liposomal amphotericin B, may reduce the sensitivity of CSF galactomannan and PCR, as fungal cell wall components and DNA are suppressed before sampling. However, it is important to note that this concern is largely extrapolated from data on serum and bronchoalveolar lavage (BAL) testing, as CSF‐specific evidence remains limited. This is especially relevant in neutropenic patients, who often receive prophylaxis or empirical therapy. Therefore, negative CSF results should be interpreted with caution in patients already on antifungals, and when possible, sampling should be pursued before therapy initiation.

Table 1 summarizes the published performance characteristics of these CSF‐based diagnostic tests, including their sensitivities, specificities, study populations, and key references.

**TABLE 1:** Performance characteristics of CSF biomarkers and molecular diagnostics for the diagnosis of CNS aspergillosis.

Test	Sensitivity	Specificity	Study population	Key reference
CSF Galactomannan (EIA)	69.0%	94.4%	Mixed immunocompromised	Komorowski et al. [22]
CSF *Aspergillus* PCR (nested)	100%	93%	Immunocompromised (HSCT, hematologic)	Reinwald et al. [24]
CSF *Aspergillus* PCR (real‐time)	75%	98.3%	Hematologic malignancies	Imbert et al. [23]
CSF mNGS	85.7%	84.6%	Suspected CNS infection	Xing et al. [27]
CSF β‐D‐Glucan	Variable	Moderate	Mixed	Mikulska et al. [26]

*Note:* This table summarizes published sensitivities and specificities of CSF galactomannan (GM) enzyme immunoassay (EIA), *Aspergillus* polymerase chain reaction (PCR) assays (nested and real‐time), metagenomic next‐generation sequencing (mNGS), and CSF β‐D‐glucan (BDG) in suspected CNS aspergillosis. Sensitivities are reported from studies that primarily included immunocompromised populations (e.g., hematopoietic stem cell transplant recipients, hematologic malignancy patients). A critical caveat, noted in the table, is that prior antifungal therapy substantially reduces the sensitivity of all CSF‐based tests, particularly GM and PCR, and negative results should not rule out disease in patients receiving empirical or prophylactic antifungal agents. Specificity remains high for most assays, supporting their rule‐in value when positive. mNGS offers the additional advantage of detecting non‐*Aspergillus* moulds and co‐pathogens.

Combining diagnostic methods, such as diffusion‐weighted MRI and CSF assays, may enhance early detection of CNS aspergillosis [25]. These findings suggest that CSF‐based diagnostics, particularly PCR and galactomannan testing, are valuable tools for diagnosing CNS aspergillosis, especially when conventional methods fail.

## 4 Antifungal Therapy: Pharmacokinetics and Clinical Data

### 4.1 CNS Penetration and Pharmacology

The efficacy of antifungal agents in CNS aspergillosis is heavily influenced by their ability to penetrate the blood–brain barrier and achieve therapeutic concentrations within the CSF and parenchyma as highlighted in Table 2.

**TABLE 2:** Pharmacokinetics and key considerations of antifungal agents for CNS aspergillosis.

Drug	CSF:plasma ratio	First‐line?	Key considerations
Voriconazole	~50%	Yes	Requires TDM; CYP450 metabolism; visual and hepatic side effects [31]
Isavuconazole	30%–50%	Alternative	Fewer CNS side effects; long half‐life; no TDM routinely needed [32]
Liposomal Amphotericin B	< 5%	Adjunctive	Poor CNS penetration; nephrotoxicity [33]
Posaconazole	~25%	Salvage	Limited CNS penetration data; useful in combination regimens [34]
Olorofim	Experimental	No	Promising CNS bioavailability; active against azole‐resistant strains [35, 36]

*Note:* Comparative overview of antifungal drugs used in CNS aspergillosis, including CSF‐to‐plasma penetration ratios, first‐line recommendations, and critical pharmacological considerations. Data highlight voriconazole as the preferred agent due to superior CNS penetration (~50%), while experimental therapies like olorofim show promise against resistant strains.

Abbreviations: CYP450, cytochrome P450 enzyme system; TDM, therapeutic drug monitoring.

Measuring drug concentrations in the CNS remains challenging. While CSF levels are most accessible, they may not accurately reflect parenchymal concentrations, particularly for lipophilic agents like voriconazole that penetrate well into brain tissue but achieve lower CSF levels. Similarly, amphotericin B and its lipid formulations have limited distribution in CSF but achieve higher concentrations within brain parenchyma, which may explain their clinical efficacy despite poor CSF penetration [37, 38]. Brain tissue sampling is rarely feasible outside of surgical resection or autopsy. Emerging microdialysis techniques and PET imaging may offer more precise pharmacokinetic data in future studies. Clinicians should be aware that CSF trough levels may underestimate therapeutic efficacy at the site of infection.

### 4.2 Treatment Algorithm

The treatment approach for CNS aspergillosis combines antifungal therapy, surgical intervention when indicated, and immunomodulation in compromised hosts (Table 3) [39, 40, 41, 42, 43]. Voriconazole remains the first‐line agent due to its superior CNS penetration and survival benefits, as demonstrated in clinical trials [44, 45]. Isavuconazole offers a safer alternative with fewer drug interactions, while liposomal amphotericin B is reserved for azole‐refractory cases or contraindications [40]. Salvage regimens, such as combination therapy (e.g., voriconazole plus an echinocandin or amphotericin B), may be considered for progressive disease, though evidence is limited to retrospective studies [39, 46]. However, combination therapy has been recently evaluated in a prospective randomized clinical trial [47], but still routine combination therapy is not recommended without refractory disease. Recent data from the ESCAI study further support isavuconazole as a safe and effective alternative, with fewer drug interactions than voriconazole [1]. Adjunctive measures, including neurosurgical debridement for abscesses > 2.5 cm and immunomodulation (e.g., interferon‐γ in chronic granulomatous disease), are critical for reducing fungal burden and improving outcomes [48]. Surgical resection, when feasible, is associated with improved survival [49, 50]. However, overall mortality remains high, especially in cases with hydrocephalus. Therapeutic drug monitoring is essential to optimize voriconazole and posaconazole levels, particularly in the CNS, where subtherapeutic concentrations can lead to treatment failure [51]. Duration of therapy is prolonged (6–12 months or longer) and tailored to clinical response, radiographic resolution, and host immunity [52].

**TABLE 3:** Treatment algorithm for CNS aspergillosis: First‐line, salvage, and adjunctive strategies.

1. First‐line antifungal therapy A. Voriconazole (preferred first‐line) Loading dose: 6 mg/kg IV every 12 h for 24 h. Maintenance: 4 mg/kg IV every 12 h (or 200 mg PO every 12 h if oral absorption is reliable). Therapeutic drug monitoring (TDM): Target trough 2–5 mg/L (adjust for CNS penetration). Key considerations: ○ Monitor for hepatotoxicity, neurotoxicity, and visual disturbances. ○ CYP450 interactions (e.g., rifampin reduces levels; SSRIs increase toxicity). B. Isavuconazole (alternative first‐line) Loading dose: 200 mg IV/PO every 8 h for 48 h. Maintenance: 200 mg IV/PO daily. Advantages: Fewer drug interactions, better tolerated than voriconazole. TDM: Not routine, but consider in CNS disease (target trough > 2 mg/L). C. Liposomal Amphotericin B (L‐AMB) (If Azoles Contraindicated) Dose: 5 mg/kg IV daily. Limitations: Poor CNS penetration (< 5%); nephrotoxicity requires monitoring.
**2. Salvage therapy (refractory/progressive disease)** A. Combination therapy Voriconazole + L‐AMB: Synergistic potential; limited robust data but often used clinically. Voriconazole + Echinocandin (e.g., caspofungin): Case reports suggest benefit, though echinocandins have minimal CNS penetration. B. Alternative azoles Posaconazole: 300 mg PO/IV daily (delayed‐release tablet preferred). TDM target trough > 1.25 mg/L. High‐dose Isavuconazole: 200 mg every 12 h (investigational, limited data). C. Experimental agents Olorofim: Available via compassionate use for azole‐resistant strains (e.g., TR34/L98H). Not yet FDA‐approved.
**3. Adjunctive interventions** A. Neurosurgical debridement/drainage Indications: Abscess > 2.5 cm, mass effect, or obstructive hydrocephalus. Goal: Reduce fungal burden and improve drug penetration. B. Immunomodulation (for immunocompromised hosts) Granulocyte transfusions: If prolonged neutropenia. Interferon‐γ (IFN‐γ): 100–150 mcg SC 3×/week (e.g., in chronic granulomatous disease). C. Corticosteroids Use cautiously: Only for severe edema/mass effect (e.g., dexamethasone 4–6 mg/day).
**4. Monitoring & duration** A. Therapeutic drug monitoring (TDM) Voriconazole/Posaconazole: Mandatory (trough levels weekly until stable). Isavuconazole: Consider in CNS disease. B. Duration of therapy Minimum: 6–12 months (or until radiographic resolution and immune recovery). Chronic Suppression: Lifelong azoles may be needed in persistently immunocompromised hosts. C. Follow‐Up Imaging MRI brain: At 2–4 weeks, then every 3 months until stability.
**5. Special considerations** Antifungal resistance testing: Perform if treatment fails (e.g., EUCAST broth microdilution for azole resistance). HIV/immunocompromised hosts: Optimize ART or reduce immunosuppression (e.g., taper steroids in transplant patients). Pediatric considerations: Pediatric patients often require higher weight‐based doses of voriconazole (e.g., 7–9 mg/kg IV q12h). Drug levels should be closely monitored due to variable pharmacokinetics and increased risk of hepatotoxicity and visual disturbances.

*Note:* Evidence‐based management approach integrating antifungal therapy, neurosurgical intervention, and immunomodulation. Voriconazole remains first‐line, with dose adjustments and TDM (target trough 2–5 mg/L) for optimal CNS penetration. Salvage options include combination therapy (e.g., voriconazole + liposomal amphotericin B) and investigational agents (e.g., olorofim). Adjunctive measures emphasize surgical debridement for abscesses > 2.5 cm and IFN‐γ in immunocompromised hosts. Duration typically extends 6–12 months, guided by imaging and immune recovery.

Abbreviations: ART, antiretroviral therapy; L‐AMB, liposomal amphotericin B.

## 5 Future Directions

### 5.1 Novel Therapeutics

The emergence of triazole‐resistant *Aspergillus* strains and limited CNS penetration of existing antifungals has spurred the development of novel agents.

- Olorofim: A first‐in‐class dihydroorotate dehydrogenase (DHODH) inhibitor with potent activity against *A. fumigatus* , including azole‐resistant strains. Unlike triazoles, it targets pyrimidine biosynthesis, reducing cross‐resistance risks. A phase 2 trial is currently evaluating olorofim in patients with invasive mould infections, including CNS disease, with preliminary results suggesting favourable safety and efficacy [53]. In vivo studies have shown promising results in murine models of invasive aspergillosis, with significant improvements in survival rates and reduction of fungal burden in both neutropenic and chronic granulomatous disease mice [35]. Olorofim's unique mechanism of action may help address concerns about antifungal resistance and provide a new treatment option for refractory infections [36, 54]. While clinical data are limited, ongoing trials are evaluating its efficacy and safety in humans [55].
- Fosmanogepix (APX001): A prodrug of manogepix is a novel broad‐spectrum antifungal agent currently in Phase 2 clinical trials for invasive fungal infections [56, 57]. It demonstrates potent activity against various *Candida* and *Aspergillus* species, including drug‐resistant strains, as well as rare moulds like Scedosporium and Lomentospora prolificans [58]. Manogepix inhibits the fungal enzyme Gwt1, involved in GPI anchor biosynthesis [59]. In vitro and in vivo studies have shown efficacy against a wide range of fungal pathogens, including those resistant to other antifungal classes [56, 57]. Clinical trials have revealed high oral bioavailability, favourable drug–drug interaction profiles, and extensive tissue distribution, making fosmanogepix an attractive option for treating invasive fungal infections [56, 57]. Ongoing research aims to further evaluate its potential in difficult‐to‐treat fungal infections [58].

### 5.2 Drug Delivery Innovations

Overcoming the blood–brain barrier remains a major challenge in CNS aspergillosis. Emerging strategies include:

- Nanoparticle/liposomal formulations: Recent research highlights the potential of lipid‐based and polymeric nanoparticle systems for improving drug delivery to the CNS. Liposomes and polymeric nanoparticles can enhance CNS delivery by bypassing efflux pumps and improving drug stability [60, 61]. These nanocarriers show promise for delivering amphotericin B (AmB), a broad‐spectrum antifungal agent with poor solubility and high toxicity [62]. Lipid‐based formulations of AmB, such as liposomal AmB, have demonstrated reduced toxicity and improved efficacy [62]. Polymeric nanoparticles, like those made from polycaprolactone, have also shown potential for AmB delivery, with high encapsulation efficiency and improved activity against Leishmania parasites compared to free AmB [63]. These advanced delivery systems offer opportunities to enhance drug targeting, stability, and release patterns, potentially improving treatment outcomes for CNS disorders [60, 61].
- Intrathecal antifungal administration: Intrathecal administration of antifungal agents has shown promise in treating refractory CNS infections. However, significant toxicities including chemical meningitis, arachnoiditis, and neurotoxicity have been documented. Consequently, the IDSA *Aspergillus* guidelines specifically do not recommend routine intrathecal therapy. In highly selected salvage cases with failure of all systemic options, it should be undertaken only at specialized centers with careful monitoring. Continuous intrathecal liposomal amphotericin B successfully treated a case of refractory *Cryptococcus neoformans* encephalitis after intravenous administration failed [64].

### 5.3 Host‐Directed Therapies

Immunomodulation is a promising adjunct, particularly in immunosuppressed patients (e.g., post‐transplant, hematologic malignancies).

- Checkpoint inhibitors (PD‐1/PD‐L1 blockade): Recent studies suggest that immune checkpoint inhibitors (ICIs) may enhance antifungal immunity. PD‐1/PD‐L1 blockade has shown promise in treating fungal infections by improving survival, reducing fungal burden, and increasing pro‐inflammatory cytokines in murine models of pulmonary aspergillosis and mucormycosis [65, 66]. Combination therapy with antifungals, such as caspofungin, demonstrated synergistic effects [65]. However, ICI therapy may also lead to immune dysregulation, potentially increasing the risk of opportunistic infections like *Listeria monocytogenes* [67]. While preclinical studies and clinical case reports support the use of ICIs as an adjunct immune enhancement strategy for fungal infections, significant knowledge gaps remain [68]. Further research is needed to determine the optimal use of ICIs in medical mycology, including the development of more nuanced animal models and studies on combination therapies, potential resistance, and the impact of the immune microenvironment [68].
- Personalized immunotherapy: CARD9 deficiency has been identified as a genetic condition that predisposes individuals to fungal infections, particularly extrapulmonary *Aspergillus* and *Candida* infections [69, 70]. This deficiency impairs the production of pro‐inflammatory cytokines and neutrophil‐recruiting chemokines, leading to inadequate neutrophil accumulation at infection sites despite normal neutrophil counts in peripheral blood [69, 71]. The CARD9 protein plays a crucial role in innate immune signalling, particularly in response to fungal pathogens [70]. Patients with CARD9 mutations exhibit reduced numbers of Th17 cells, which are important for antifungal immunity [70]. The host defence against *Aspergillus* involves a complex network of cytokines, with TNF initiating the innate response and Th1‐associated cytokines (IL‐12, IL‐18, IFN‐gamma) providing protection, while Th2 cytokines (IL‐4, IL‐10) contribute to infection progression [71, 72]. Advances in host genomic profiling (e.g., *CARD9* mutations) and cytokine modulation (e.g., IFN‐γ, GM‐CSF) may guide tailored therapy.

## 6 Conclusion

CNS aspergillosis remains a rare but devastating disease with persistently high mortality rates. Between 2020 and 2026, notable progress has been made in AI‐aided diagnostics, molecular assays, and antifungal pharmacotherapy. A tailored, multidisciplinary approach combining imaging, targeted therapy, surgical intervention, and immune modulation offers the best chance at improving outcomes.

## Author Contributions

**Mohamed A. S. El Molla:** funding acquisition, writing – review and editing, project administration. **Mohamed H. Helmy:** funding acquisition, writing – review and editing, project administration. **Hamdi Nabawi Mostafa:** conceptualization, investigation, writing – review and editing, project administration, supervision. **Taha Mohammed Adel:** funding acquisition, writing – review and editing, formal analysis. **Moustafa A. Mansour:** conceptualization, investigation, writing – original draft, methodology, writing – review and editing, software, project administration, formal analysis, data curation, supervision, resources. **Mohamed Wahid:** funding acquisition, writing – review and editing, project administration.

## Funding

The authors have nothing to report.

## Ethics Statement

The authors have nothing to report.

## Consent

The authors have nothing to report.

## Conflicts of Interest

The authors declare no conflicts of interest.

## Data Availability

All data generated or analysed during this study are included in this published article.
